# Evaluating the Effect of Different Polymer and Composite Abutments on the Color Accuracy of Multilayer Pre-Colored Zirconia Polycrystal Dental Prosthesis

**DOI:** 10.3390/polym14122325

**Published:** 2022-06-08

**Authors:** Wen-Chieh Hsu, Tzu-Yu Peng, Chien-Ming Kang, Fan-Yi Chao, Jian-Hong Yu, Su-Feng Chen

**Affiliations:** 1 School of Dentistry, College of Dentistry, China Medical University, Taichung 40402, Taiwan; hsu.alic88@msa.hinet.net; 2 School of Dentistry, College of Oral Medicine, Taipei Medical University, Taipei 11031, Taiwan; typeng@tmu.edu.tw; 3 Huayi Dental Laboratory, Taipei 10491, Taiwan; kjm670815@hotmail.com; 4 Department of Physiology and Biophysics, University of Colorado School of Medicine, Aurora, CO 80045, USA; xeriod033363@gmail.com

**Keywords:** spectrophotometry, optical properties, digital dentistry, CAD/CAM, 3D printing, composite resin, polyetheretherketone, polyetherketoneketone, monolithic zirconia polycrystal

## Abstract

With increasing aesthetic awareness and emphasis on time costs in today’s society, monolithic multilayer precolored zirconia ceramics (M-Zr) facilitate aesthetic restorations in a convenient and straightforward manner without the need for veneering porcelain to modify the color. However, the effect of abutment materials on the final color of M-Zr remains unclear. Herein, we placed Vita A1 Shade M-Zr on six different abutment materials, zirconia (Y-TZP), 3D printed composite resin (CR), dental model resin (MR), polyetheretherketone (PEEK), polyetherketoneketone (PEKK), and cobalt–chromium alloy (Co–Cr), to evaluate their effect on the color accuracy of M-Zr. The color attributes (L*, a*, and b*) were measured using a dental spectrophotometer. The translucency parameter (TP), contrast ratio, color difference (ΔE) between each background substrate and the Vita A1 Shade Guide, and chroma values (C) were calculated to evaluate the color accuracy of M-Zr. A statistical analysis was performed using one-way analysis of variance and post hoc Tukey’s HSD tests (α = 0.05). The experimental results indicate that the TP values and contrast ratio of the M-Zr samples were 14.85 and 0.83, respectively. Co–Cr had the highest ΔE (6.08) and lowest C value (7.52); PEKK had the lowest ΔE (2.60), and PEEK had the highest C value (12.23) (*p* < 0.05). Notably, the ΔE values of CR (3.13), PEEK (2.86), and PEKK were within clinical indicators (ΔE < 3.7). Based on these results, it can be concluded that the abutment material has a significant effect on the final color of the M-Zr, and PEEK or PEKK resulted in good color accuracy. When choosing the dental MR, traditional zirconia, or metals as abutment materials, colored or opaque cement might be required to eliminate color distortion and achieve desirable optical properties.

## 1. Introduction

Currently, digital dentistry is the mainstream of dentistry. Through intraoral scanning, impression images can be quickly and accurately obtained reducing the discomfort caused by the traditional impression and speeding up the treatment [1,2]. Digital data obtained from intraoral scanners can be quickly transmitted through computers and the internet. The dental laboratory receives the files; it can, through a computer-aided design and computer-aided manufacturing (CAD/CAM) system, design, model, and manufacture the dental prosthesis via 3D printing or milling [3,4,5,6]. CAD/CAM has become an indispensable part of the dentistry, as it improves precision, reduces the time cost, and increases reproducibility [6,7]. Moreover, in modern society, regardless of the age group, the demand for aesthetics is increasing day by day [7,8]. Traditional dental prostheses use metals or alloys as the base materials and build up the feldspar-based ceramics to achieve aesthetics and functionality. However, with the increasing price of precious metals and concerns about metal allergies, metal-free treatment has become a trend in dentistry [9,10].

Many 3D printing biocompatible resins and polymer materials have become available for dental use in recent years. By adjusting the ratio of an inorganic filler and resin, composite resin (CR) with sufficient strength, opacity, translucency, and pleasing aesthetics can be created. A CR-printed prosthesis can blend perfectly between the existing teeth by simply finishing and polishing [11,12]. It is often used as a temporary crown, dental core, or coping material during dental treatment. The emergence of the model dental resin (MR) is particularly significant for dental clinics because it can omit the complicated modeling and pouring process and directly print a diagnostic model through a 3D printer, which saves time and is clean and hygienic [13]. Polyaryletherketone (PAEK) is a family of novel high-performance semicrystalline thermoplastic polymers consisting of an aromatic backbone molecular chain interconnected by ketone (C=O) and ether (C–O) functional groups [14,15]. Many polymers, which are based on different orders and ratios of functional groups, are included in the PAEK family. The most well-known dental PAEK polymers are polyetheretherketone (PEEK) and polyetherketoneketone (PEKK) [15,16]. PAEK has excellent biocompatibility, chemical stability, and radiolucency and can be manufactured through milling or 3D printing based on the clinical needs for the customization of dental prosthesis [11]. In addition, PAEK is characterized by a low density, several color options, and an elasticity modulus similar to that of human hard tissue. Therefore, PAEK as an abutment material (e.g., dental core or coping) exhibits shock absorption and raises no concern on the problem of color penetration [16,17].

Moreover, the near-bioinert ceramic material, “polycrystalline zirconia ceramic,” is an attractive aesthetic dental material owing to its excellent mechanical strength, biocompatibility, and appearance [18]. Monolithic zirconia is a new type of polycrystalline zirconia ceramic with properties comparable to those of traditional zirconia; however, monolithic zirconia does not require veneering porcelain to modify the color appearance, thereby preventing the chipping of the porcelain layer [19]. The failure rate of monolithic zirconia restorations in the 5-year follow-up survey was only 2.6%, which is lower than that of any other ceramic restorative material [20]. However, the color performance of the monolithic zirconia restoration itself has a considerable impact on whether the final color of the prosthesis matches the natural tooth [21]. With rapid progress in material science, monolithic zirconia restorations can be precolored on porous zirconia blocks during manufacturing. Such zirconia is called “monolithic multilayer precolored zirconia polycrystals (M-Zr)”. M-Zr has a natural color gradation from dentin (opaque) to the incisal edge (transparent) and fluorescence, suitable for aesthetic areas. In addition, working with M-Zr is fast and straightforward for both the chair side and the lab side. The variation factor of human operation is low and has high stability. Thus, it is the best choice in aesthetic material for restorative dentistry [22,23,24,25].

Kim et al. [26] discussed the color performance of M-Zr adjusted by the coloring liquid. Sulaiman et al. [27] and Tabatabaian et al. [28] studied the effect of the thickness of M-Zr on its final color. Kang et al. [23] proposed that various types of porous zirconia blocks and their different transparencies affect its final color; all these factors should be considered to achieve optimal aesthetics. However, these studies have not considered the influence of different color substrates (i.e., abutment materials), such as dental copings or cores, implant abutments, and abutment teeth, on the final color of M-Zr. In dental clinics, the tooth fabrication on 3D printed models (lab side) is often done by dental technicians, and the dentist selects various abutment materials in accordance with the patient’s oral condition (chair side). However, the color of the substrate affects the final color of the dental restoration material, leading to color differences between the chair side and lab side. If this distortion can be avoided, the time and cost of the clinic and the patient can be reduced. This study aims to investigate the color accuracy of M-Zr when different substrates (abutment materials) are used. It is hoped that some primary data can be established for the future clinical use of M-Zr as a reference to address the cognitive differences between dentists and technicians. Furthermore, it can reduce the number of return appointments and increase patient satisfaction. The null hypothesis of this experiment is that the color of M-Zr dental prostheses is not dependent on the type of abutment material.

## 2. Materials and Methods

Six different substrates (abutment materials) (Table 1) and a gray background were considered for this experiment. The substrates included two dental 3D printing resins (CR and MR), two semicrystalline thermoplastic polymers (PEEK and PEKK), one zirconia polycrystal, and one cobalt–chromium alloy. All substrates were designed with 10 × 10 mm size and 2 mm thickness via Solid-Works software (Dassault Systèmes SolidWorks, Waltham, MA, USA). Dental resins were 3D printed by Phrozen Sonic XL 4K (Phrozen Tech Co., Ltd., Hsinchu, Taiwan). The thermoplastic polymers and zirconia polycrystals were fabricated using milling by a dental CAD/CAM system (Cameo 250i; Aidite Technology Co., Ltd., Qin Huang Dao, China). The Co–Cr alloy was 3D printed by Riton Laser D-100 (Rxton Technology Co., Ltd., Guangzhou, China). A standardized professional photography card (QP Card 101; QPcard AB, Göteborg, Sweden) was used for the gray background.

**Figure 1:**
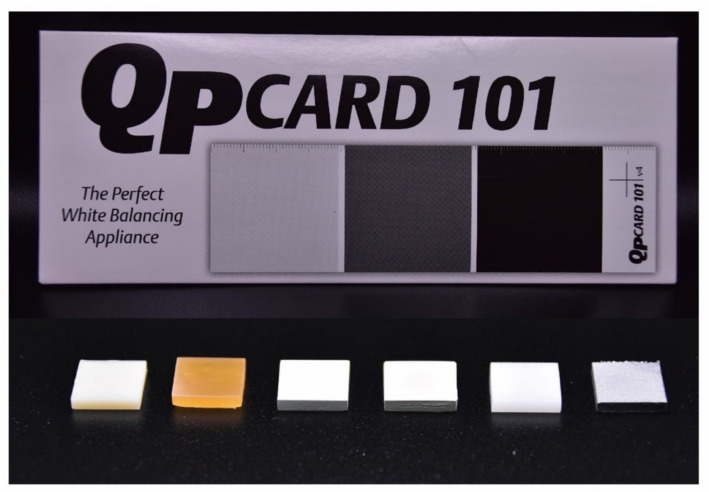
Substrate material. The upper row is the QP Card (white, gray, and black); the lower row shows CR, MR, PEEK, PEKK, Y-TZP, and Co–Cr in that order.

An A1 shade (Vita Zahnfabrik, Bad Säckingen, Germany) monolithic multilayer precolored zirconia polycrystal (3D Pro Multilayer; Aidite Technology Co., Ltd., Qin Huang Dao, China) was used for testing dental crown materials (Table 1). The test samples were plate-shaped with dimensions of 8 × 8 mm and a thickness of 1 mm. The samples were produced using a dental CAD/CAM system (Cameo 250i; Aidite Technology Co., Ltd., Qin Huang Dao, China) without any staining, polishing, or coloring process. Prior to the measurements, all the samples were ultrasonically cleaned in distilled water for fifteen minutes and air dried. Figure 1 illustrates the substrate materials and test samples used in this experiment.

**Figure 2:**
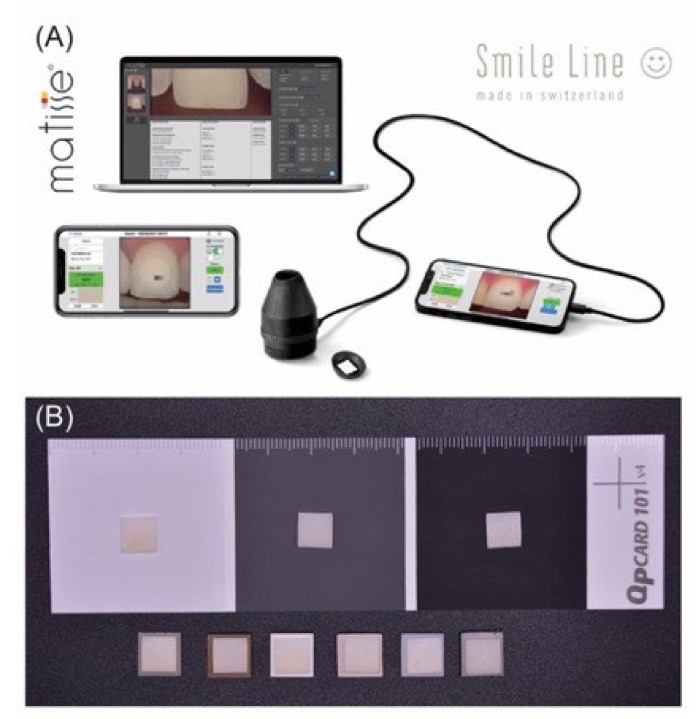
(**A**) Schematic of optical measurement and (**B**) the appearance of the testing sample placed on different substrates.

Optical measurements were performed using a dental spectrophotometer (Optishade Styleitaliano; Smile Line SA, St-Imier, Switzerland). All measurements were performed by the same operator under the same conditions to exclude errors arising from variations among humans. The spectrophotometer was calibrated after every ten measurements. Three color attributes, L* (lightness coordinate), a* (red–green coordinate), and b* (yellow–blue coordinate), were obtained according to the Commission Internationale de l’ E-clairage (CIELAB) system. First, five different substrate materials (CR, MR, PEEK, PEKK, Y-TZP, and Co–Cr) were placed on a transparent background (using a clear acrylic sheet) to measure the color attributes. Then, the color attributes (L*, a*, and b*) of the M-Zr samples (n = 10) were measured by placing them on different substrates (Figure 2).

The translucency parameter (TP) values describe the masking ability of the samples, and the contrast ratio estimates the opacity of the samples. The TP values and contrast ratios were determined by calculating the color difference of the specimen when placed over the black and white substrates using the formula [29,30] depicted in Equations (1) and (2), where the subscripts *B* and *W* refer to color coordinates when M-Zr is placed over black and white substrates, respectively. The black and white substrates used were standardized professional photography cards (QP Card 101; QPcard AB, Göteborg, Sweden).

(1) $$TP=\sqrt{{\left(L_{B}^{*}-L_{W}^{*}\right)}^{2}+{\left(a_{B}^{*}-a_{W}^{*}\right)}^{2}+{\left(b_{B}^{*}-b_{W}^{*}\right)}^{2}}$$

(2) $$contrast\;ratio=\frac{L_{B}}{L_{W}}$$

Color difference (ΔE) between the measured color attribute of M-Zr on each substrate and the Vita A1 Shade guide (L* = 76.7, a* = 1.1, and b* = 14.7) under each substrate were calculated using the following CIEDE2000 color difference formula [31] depicted in Equation (3).

(3) $$\Delta E=\sqrt{{\left(\frac{\Delta L\prime }{k_{L}\;S_{L}}\right)}^{2}+{\left(\frac{\Delta C\prime }{k_{C}\;S_{C}}\right)}^{2}+{\left(\frac{\Delta H\prime }{k_{H}\;S_{H}}\right)}^{2}+R_{T}\left(\frac{\Delta C\prime }{k_{C}\;S_{C}}\right)\left(\frac{\Delta H\prime }{k_{H}\;S_{H}}\right)}$$

where Δ*L*′, Δ*C*′, and Δ*H*′ are the differences in lightness, chroma, and hue, respectively; *k_L_*, *k_C_*, and *k_H_* are weighting factors for lightness, chroma, and hue, respectively; *S_L_, S_C_*, and *S_H_* are averaging factors for lightness, chroma, and hue, respectively; *R_T_* is an overall correction factor based on differences in hue and chroma.

Chroma refers to the purity or saturation of color and is determined by the ratio of a certain solid color; the chroma (C) values calculated herein are the degree of color departure from the neutral color of the same value. The C value can be calculated under a gray substrate using the following formula [32], denoted by Equation (4).

(4) $$C=\sqrt{{\left(a^{*}\right)}^{2}+{\left(b^{*}\right)}^{2}}$$

The data obtained were recorded, and statistical analyses were performed. Normality and homogeneity were primarily analyzed using Shapiro–Wilk and Levene’s tests. All data indicated a normal distribution and homogeneity; therefore, parametric tests were used in the current experiment. The C values were analyzed using one-way analysis of variance (ANOVA) followed by the post hoc Tukey honestly significant difference (HSD) test. The differences in the color attributes (L*, a*, b*, and ΔE) detected on different substrates were analyzed with one-way repeated measures ANOVA and post hoc comparisons using the Tukey HSD test. All statistical analyses were performed using Prism 9.0 (GraphPad Software, Inc., La Jolla, CA, USA), and differences between each variant were considered significant at *p* < 0.05.

## 3. Results

The color attributes (L*, a*, b*) of the six different substrate materials (CR, MR, PEEK, PEKK, Y-TZP, and Co–Cr) are listed in Table 2. PEEK had the highest L* value (90.90), and MR had the highest a* (12.73) and b* (36.57) values. Co–Cr had the lowest L* value (31.97); CR had the lowest a* value (−2.23), and Y-TZP had the lowest b* value (−0.13). The mean and standard deviations for the color attributes of the M-Zr samples detected on different substrates are shown in Table 3. When compared against three professional photography QP cards (black, white, and gray), all color attributes exhibited the highest value on the white card (*p <* 0.05). For the other substrates, the L* value of PEEK was the highest (79.99) and that of Co–Cr was the lowest (72.28); the a* value of MR was the highest (2.53) and that of CR was the lowest (−0.47); the b* value of PEEK was the highest (12.15) and that of Co–Cr was the lowest (7.52).

**Figure 3:**
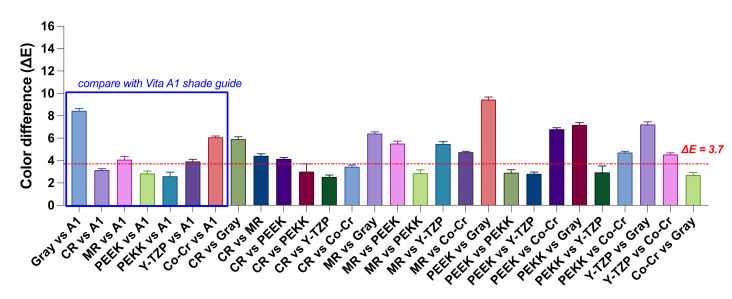
Pairwise comparison of the color difference (ΔE) of M-Zr samples measured under different substrates. Blue box represented the ΔE compared with the Vita A1 Shade guide (L* = 76.7, a*= 1.1, and b* = 14.7). Horizontal dotted line represents clinical indicators of ΔE = 3.7 [33].

The TP values and contrast ratio of the M-Zr samples were 14.85 and 0.83, respectively. Figure 3 shows the color difference (ΔE) among the M-Zr samples when measured on different substrates. The literature [33] suggests that the average acceptable color difference between two teeth under comparison to be considered as a match in the oral environment is 3.7. When the groups were compared using Vita A1 Shade guide (blue box in Figure 3), the ΔE values of CR (3.13), PEEK (2.86), and PEKK (2.60) were within the clinical indicators (ΔE <3.7). Meanwhile, post hoc Tukey HSD tests revealed that there is no significant difference among the three groups—Y-TZP and MR, CR and PEEK, and PEEK and PEKK (*p* > 0.05).

**Figure 4:**
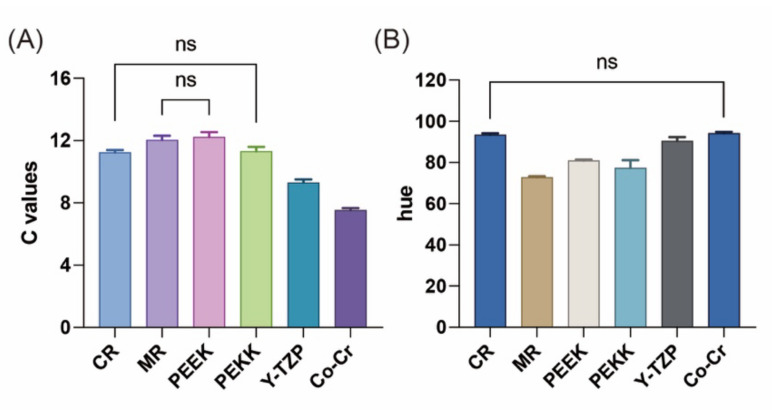
Results of average (**A**) chroma (C) values and (**B**) hue (h) under different substrate groups. No statistical differences between groups are shown as “ns”.

Figure 4 illustrates the C value and hue for the M-Zr samples for different substrate groups. The C values of the two 3D printing resins CR and MR and those of the thermoplastic polymers PEEK and PEKK were higher than those of Y-TZP and Co–Cr. The post hoc Tukey HSD test indicated that a significant difference appeared not only for CR and PEKK but also for MR and PEEK (*p <* 0.05). All the hue values ranged from 72.78 to 100.52, and no significance was found in CR and Co–Cr (*p <* 0.05).

## 4. Discussion

M-Zr is expected to mimic the transparency and strength of human dentin and enamel while avoiding the possibility of delamination or chipping of bilayer material restorations [34,35]. Factors affecting the color of M-Zr restorations include the manufacturing processes, laboratory procedures, and clinical factors [36]. Kang et al. [22,23] evaluated the effects of the manufacturing processes and laboratory procedures on the color accuracy of M-Zr. However, clinical factors that include abutment material and cement color also affect the resulting color. According to the results of this study, when the thickness (1.0 mm) and the initial color (Vita A1 Shade) of M-Zr is maintained constant, the final color accuracy will depend on the type of abutment material; hence, the null hypothesis was rejected.

Numerous researchers have discussed the effect of thickness on the optical properties of monolithic zirconia [22,23,28,37,38,39]; however, a previous study declared that the minimum thickness of M-Zr should be 1.0 mm to achieve the best color accuracy [22]. Hence, a thickness of 1.0 mm was selected for the test specimens in this study. According to the literature, a high transparency of zirconia would cause scattering, which in turn would influence the resultant color because of the background, affecting color accuracy [40].

Metals have long been used in dentistry owing to their good mechanical properties [9]. Polycrystalline zirconia ceramic materials have also been widely used in recent years owing to their excellent biocompatibility [41]. These two types of materials are considered as traditional abutment materials in this experiment; therefore, the author chose Co–Cr and Y-TZP as their representatives. Co–Cr is a metal with low brightness (Table 2) and when M-Zr is placed upon it, its C (Figure 4) and L* values (Table 3) reduce further, resulting in a turbid color. This kind of abutment material results in a color difference of 6.08 and makes it difficult to modify the color even with porcelain veneer staining or glazing adjustment. In addition, a low b* value makes the base color bluer (Table 3) and affects the final color accuracy. Therefore, if a metal abutment material is used clinically, the color of the abutment teeth can only be blocked by using cement with a color-shielding effect (such as an opaque cement) in order to achieve color accuracy at the cost of compromising excellent transparency and tooth-like color of M-Zr materials. Y-TZP used in this experiment is a white-shaded single layer with high translucency; therefore, it has a high L* value (79.40), resulting in a whiter overall material (Table 2). The white shade of pure Y-TZP leads to a lower C value (Figure 4). However, Y-TZP has good transparency (48%); therefore, when it is used as an abutment material with M-Zr placed upon it, it exhibits good light transmittance, and the color difference would be only slightly higher than that of the clinical indicators (ΔE = 3.93) [33]. Thus, when clinically using white-shaded zirconia as an abutment material, it is recommended to choose cement with bleach or tooth (e.g., A1, A2, etc.) color to slightly reduce the influence of white zirconia on the optical properties of M-Zr, thereby improving color accuracy.

Three-dimensional printing has the advantages of convenience, low pollution, and fewer residual materials [3,4]. In the field of dentistry in recent years, CR, MR, and acrylic resin have been used widely for making temporary crowns, working models, and removable prostheses, respectively. In this current study, Vita A2 shade CR was used to mimic human teeth. In addition, a yellowish-orange-colored MR, which is widely used in the preparation of a working model, was included. Because the optical properties of the CR itself are close to those of the teeth, the results of various color attributes are close to clinical indicators and have good chromaticity and a low color difference (ΔE = 3.13). Therefore, when CR is used as an abutment material, there is no need to consider the influence of background color on M-Zr; therefore, the choice of cement does not arise. Some pigments are added to MR, which makes its color yellowish-orange (Figure 1), resulting in higher a* and b* values (Table 2), which further causes the color of M-Zr on the MR to have higher a* and b* values (Table 3). Moreover, the C value also increases (Figure 4), while it also exhibits lower brightness, which was only slightly higher than that of Co–Cr (Table 3). Previous studies have indicated that when coloring pigments increase, the chroma values increase, but brightness decreases, and the color difference increases [26,42]. The results of the current experiment also confirm that the MR has a higher ΔE ((4.08) than the clinical indicator of ΔE < 3.7; therefore, it is recommended to use a cement that is similar to that of Y-TZP.

Many studies have pointed out the potential of PAEK materials in dental applications, as their mechanical properties are close to those of human hard tissue and bone making them a good substrate for dental restorations and teeth [14,17]. Peng et al. [43] evaluated the antibacterial effect of PAEK materials by testing their ability to form a biofilm. The results showed that PAEK did not easily generate biofilms. Therefore, we believe that PAEK is a good abutment material. The PEEK used in this experiment is of standard white color, which can be seen from the L* value (90.90), which is highest among the substrate materials (Table 2). A little pigmentation was added to the PAEK materials to make their color appear natural. The results of various color attributes show that the PAEK materials have a better color balance (Table 3), higher color saturation, and lower hue (Figure 4) compared to those of the other groups. The above results make it possible to achieve the highest color accuracy (ΔE < 2.9) without affecting the optical properties of M-Zr when PAEK is selected as the abutment material. Therefore, the clinical use of PAEK does not raise concerns about affecting the optical properties of M-Zr.

At this stage, using CR or PAEK as the abutment materials was deduced to have the best color accuracy among all the specimens tested in this study; however, the authors only tested the Vita A1 shade M-Zr to evaluate color accuracy. Different results could be obtained when different colors of M-Zr are chosen. The color of dental cement is also a key factor affecting the final color appearance of M-Zr restorations. Therefore, these factors should be included in future studies to establish clinical guidance for the use of M-Zr.

## 5. Conclusions

Within the limitations of this in vitro study, it was possible to declare that in the clinical application of M-Zr, prior assessment of the abutment materials helped achieve good aesthetics. The transparency of M-Zr causes the color of the abutment material to have a significant effect on the final color appearance. The selection of CR, PEEK, and PEKK as abutments can lead to a better color accuracy for M-Zr. Note that when using MR or Y-TZP to make M-Zr restorations, some colored cement must be considered to reduce the color difference. However, when choosing Co–Cr, opaque cement may be required to achieve good optical properties and eliminate color distortion.

## Figures and Tables

**Table 1:** Detail of the materials used. * 4Y-PSZ, 4 mol% yttria-partially stabilized zirconia; 5Y-PSZ, 5 mol% yttria-partially stabilized zirconia

Materials	Main Composition	Manufacturer	Manufacturing Process	Code
**Substrate materials**				
***Dental 3D printing resin***				
NextDent C&B MFH	methacrylic oligomers, phosphine oxide, microfiller	NextDent B.V, Soesterberg, The Netherlands	3D printing	CR
DENTAL MODEL	aromatic methacrylic oligomer, aliphatic methacrylic oligomer, phosphine oxide	Enlighten Materials Co., Ltd., Taipei, Taiwan	3D printing	MR
***Semicrystalline thermoplastic polymers***				
VESTAKEEP	poly(ether-ether-ketone)	Evonik Japan Co., Tokyo, Japan	milling	PEEK
Pekkton ivory	poly(ether-ketone-ketone)	Cendres+Métaux SA, Biel/Bienne, Switzerland	milling	PEKK
***Zirconia polycrystal***				
Super High Translucent Plus White Zirconia	zirconium dioxide, yttrium oxide	Aidite Technology Co., Ltd., Qin Huang Dao, China	milling	Y-TZP
***Metal alloy***				
C02 (CoCrMo Powders)	cobalt, chromium, molybdenum	Material Technology Innovations Co., Ltd., Guangzhou, China	3D printing	Co–Cr
**Testing materials**				
3D Pro Multilayer (4Y-PSZ + 5Y-PSZ)*	zirconium dioxide, yttrium oxide	Aidite Technology Co., Ltd., Qin Huang Dao, China	milling	M-Zr

**Table 2:** The color attributes (L*, a*, and b*) of six different substrate materials (n = 10).

Substrate Material	L*	a*	b*
CR	71.40 ± 0.10	−2.23 ± 0.12	14.43 ± 0.12
MR	48.57 ± 0.12	12.73 ± 0.06	36.57 ± 0.15
PEEK	90.90 ± 0.01	0.73 ± 0.06	5.97 ± 0.15
PEKK	79.33 ± 0.06	3.53 ± 0.12	11.83 ± 0.06
Y-TZP	79.40 ± 0.17	−1.83 ± 0.06	−0.13 ± 0.06
Co-Cr	31.97 ± 0.12	1.71 ± 0.12	2.97 ± 0.06

All values were measured on a transparent background and are presented as mean ± standard deviations.

**Table 3:** Mean ± standard deviations (SD) for the color attributes (L*, a*, and b*) of M-Zr samples on different substrates (n = 3).

Substrate	L*	a*	b*
Black	67.91 ± 0.31	−0.86 ± 0.04	5.99 ± 0.12
White	81.62 ± 0.24	0.77 ± 0.26	11.46 ± 0.17
Gray	69.09 ± 0.30	−0.79 ± 0.06	6.30 ± 0.20
CR	75.30 ± 0.33	−0.47 ± 0.09	11.23 ± 0.14
MR	72.65 ± 0.51	2.53 ± 0.10	11.77 ± 0.26
PEEK	79.99 ± 0.28	1.35 ± 0.10	12.15 ± 0.30
PEKK	76.16 ± 0.26	1.73 ± 0.55	11.16 ± 0.26
Y-TZP	78.20 ± 0.23	0.05 ± 0.21	9.30 ± 0.21
Co-Cr	72.28 ± 0.20	−0.37 ± 0.07	7.52 ± 0.14

Black, white, and gray used QP Card 101; CR-3D printed composite resin; MR-3D printed model resin; PEEK—polyetheretherketone; PEKK—polyetherketoneketone; Y-TZP—yttria-tetragonal zirconia polycrystal; and Co–Cr—cobalt–chromium.

## Data Availability

Not applicable.
